# Healthcare professionals’ experiences of delivering a stroke Early Supported Discharge service – An example from Ireland

**DOI:** 10.1177/02692155231217363

**Published:** 2023-12-06

**Authors:** Elaine O Connor, Eamon Dolan, Frances Horgan, Rose Galvin, Katie Robinson

**Affiliations:** 1 School of Allied Health, Faculty of Education and Health Sciences, Ageing Research Centre, Health Research Institute, University of Limerick, Castletroy, Limerick, Ireland; 2 Connolly Hospital, Blanchardstown, Dublin, Ireland; 3 School of Physiotherapy, Royal College of Surgeons in Ireland (RCSI) University of Medicine and Health Sciences, Dublin, Ireland

**Keywords:** Early Supported Discharge, stroke, healthcare professionals, qualitative study

## Abstract

**Objective:**

To explore healthcare professionals’ experiences of the development and delivery of Early Supported Discharge for people after stroke, including experiences of the COVID-19 pandemic.

**Design:**

Qualitative descriptive study using one-to-one semi-structured interviews. Data were analysed using reflexive thematic analysis.

**Setting:**

Nine Early Supported Discharge service sites in Ireland.

**Participants:**

Purposive sampling identified 16 healthcare professionals.

**Results:**

Five key themes were identified (1) Un-coordinated development of services, (2) Staff shortages limit the potential of Early Supported Discharge, (3) Limited utilisation of telerehabilitation post COVID-19 pandemic, (4) Families need information and support, and (5) Early Supported Discharge involves collaboration with people after stroke and their families.

**Conclusions:**

Findings highlight how Early Supported Discharge services adapted during the COVID-19 pandemic and how gaps in the service impacts on service delivery. Practice implications include the need to address staff recruitment and retention issues to prevent service shortages and ensure consistent access to psychology services. Early Supported Discharge services should continue to work closely with families and address their information and support needs. Future research on how telerehabilitation can optimally be deployed and the impact of therapy assistants in Early Supported Discharge is needed.

## Introduction

Stroke is the second leading cause of death worldwide with 12·2 million incident cases of stroke and 6·55 million deaths from stroke in 2019.^1^ Stroke is a leading cause of disability and morbidity with an estimated €27 billion spent yearly by the European Union on stroke treatment and care.^2^ Early Supported Discharge facilitates people who have had a stroke to be discharged home from hospital earlier than otherwise possible, with rehabilitation in their own home under the care of specialist healthcare professionals.^3^ Early Supported Discharge has demonstrated a reduction in the length of hospital stay, long-term dependency and admission to residential care at 6 months among those who received Early Supported Discharge when compared to usual care.^3^

A recent qualitative evidence synthesis found that people after stroke, carers, and healthcare professionals report largely very positive experiences of Early Supported Discharge.^4^ Healthcare professionals emphasised the importance of various organisational and interprofessional factors related to the model of care, eligibility criteria, person-centred discharge planning, and interprofessional teamwork.^4^

The recently updated National Clinical Guideline for Stroke for the United Kingdom and Ireland^5^ recommends that Early Supported Discharge teams should include a co-ordinated multidisciplinary team with a minimum composition of; physiotherapy (1 WTE), occupational therapy (1 WTE), speech and language therapy (.4 WTE), social worker (up to .5 WTE), rehabilitation assistant (1 WTE), clinical psychology/neuropsychologist (.2-.4 WTE), nursing (up to 1.2 WTE), and medicine (.1 WTE) (WTE per 100 referrals to the service per annum). In Ireland, Early Supported Discharge team availability has increased in recent years from availability in three hospitals in 2013 to nine hospitals in 2020 with plans for 21 teams to be fully commissioned under the National Stroke strategy 2022–2027.^6^ However, despite these ambitious plans no hospital in Ireland in 2021 had a fully resourced team and heterogeneity in the duration of Early Supported Discharge, geographical parameters for access to the service and use of telerehabilitation across sites has been noted.^7^

In England where Early Supported Discharge is an available model of care,^8^ qualitative research with healthcare professionals has been identified as key to investigating adherence to evidence-based standards and the delivery of effective services in practice.^9^ Two qualitative studies^10,11^ report the Early Supported Discharge experiences of people after stroke in Ireland however no study to date has reported the experiences and perspectives of healthcare professionals in Ireland of the relatively recent development and delivery of this model of care. Given that organisational and interprofessional factors are seen by healthcare professionals^4,12^ as central to the success of Early Supported Discharge,^4^ there is a need to understand the delivery of Early Supported Discharge in Ireland from the perspective of the healthcare professional, particularly in the context of recent service expansion and the COVID-19 pandemic when healthcare services underwent significant reconfiguration. This study aimed to explore healthcare professionals’ experiences of the development and delivery of Early Supported Discharge stroke services in Ireland.

## Methods

The reporting of this qualitative study adhered to the Consolidated Criteria for Reporting Qualitative Research (COREQ)^13^ with a qualitative descriptive approach employed.

A purposive approach to sampling was adopted as it allowed intentional selection of participants who met the following criteria and were therefore judged to have the knowledge and relevant experience to address the research question^14^

1. A healthcare professional;
2. Involved in the delivery of Early Supported Discharge in Ireland, previously worked in stroke Early Supported Discharge in Ireland or involved in the provision of support and input to those in receipt of Early Supported Discharge but were not considered to be a designated member of the Early Supported Discharge team.

We aimed to recruit at least one healthcare professional from all nine Early Supported Discharge teams across Ireland. Several strategies were used in tandem to recruit healthcare professionals namely; a study advertisement on X (formerly known as Twitter), contacting healthcare professionals known to the research team to circulate information to healthcare professionals working in Early Supported Discharge, and distribution of recruitment materials to all nine Early Supported Discharge sites via a gatekeeper, the national Health and Social Care Professionals representative for Early Supported Discharge.

Conceptual debate and uncertainty have surrounded choosing an appropriate sample size in qualitative research.^15^ Data saturation or “information redundancy” is referenced widely in thematic analysis.^16^ We considered information redundancy during preliminary analysis of the data in tandem with data collection. Recruitment ceased once at least one participant from all nine sites was recruited and no new themes were identified with adequate information power achieved.^17^

Ethics approval was granted by the Faculty of Education and Health Sciences Research Ethics Committee at the University of Limerick [2021_12_06_EHS (ER)]. All participants were provided with an information leaflet which outlined the purpose of the study, risks and benefits and right to withdraw. A consent form was signed by participants prior to participating.

### Data collection and analysis

Data were collected via one-to-one semi-structured recorded interviews on Microsoft Teams as it allowed social distancing to be adhered to as all participants worked within the hospital environment. Only one interview was conducted with each participant with all interviews taking place in the workplace between February and June 2022. Notes were made after each interview outlining researcher reflections. The primary author (EOC) conducted all the interviews and had no prior relationship with the participants.

An interview topic guide was developed informed by a recent qualitative evidence synthesis on stakeholders’ experiences of Early Supported Discharge after stroke.^4^ Key topics reported in the evidence synthesis and included in the topic guide were; the model of care (referral pathways, eligibility criteria, team composition, intensity of therapy, interventions and services provided, discharge criteria/process), connections between Early Supported Discharge services and acute and community services, unmet service user and caregiver needs. The topic guide also addressed adaptation of services during the COVID-19 pandemic, and experiences of service developments.

The topic guide was refined after initial interviews were completed to include topics emerging from the interviews that were unanticipated by the researchers and had not been explored with other participants, for example people after stroke with subtle hidden impairments as an emerging cohort. All interviews were conducted by EOC, a female occupational therapist and PhD student. EOC completed training in qualitative research as part of her postgraduate studies which was aimed at early-career researchers. A diary was maintained throughout the study to reflect on ideas and assumptions following each interview and throughout data analysis to consider her impact on the research process. Discussions with supervisors (KR and RG) supported critical self-awareness of the lead researcher throughout data collection and analysis (EOC). Though the lead researcher did not work during or prior to data collection in Early Supported Discharge, the commitment to reflexivity was important given the lead researchers background as an occupational therapist and experiences of working in rehabilitation and enhances the rigour of the study.^18^

Audio recordings were transcribed verbatim by EOC. All participants were assigned pseudonyms mapping to occupation. Clinical experience amongst participants ranged from 2.5 years to 22 years. All transcripts were imported into NVivo software package (Version 12 QSR International) for analysis. These transcripts were not returned to participants for comment or correction. Braun and Clarke's^19^ six phases of reflexive thematic analysis were applied. Phase one involved immersion through reading and re-reading the transcripts and documenting initial thoughts and ideas. An inductive approach to coding was adopted which involved being specific and detailed in capturing concepts related to the research aim in the data. During this phase, the primary author (EOC) initially coded two sets of transcripts and were discussed and reviewed in detail with KR before completing the coding of the remaining transcripts. In the third phase, all the codes were reviewed and discussed with provisional themes identified. Similar codes were collated which assisted the primary author in generating themes which were descriptive rather than richly interpretative of the data. Phase four involved returning to reviewing the transcripts and codes to ensure that these fit the provisional themes. Phase five involved the writing up of the themes ensuring that the key aspects of the theme were captured. This phase included editing and refining the themes. In the final phase, further writing and editing occurred, and choosing what excerpts from the transcripts to reflect the theme.

## Findings

Sixteen healthcare professionals across nine stroke specific Early Supported Discharge sites in Ireland consented to participate and were interviewed; one clinical nurse specialist, one consultant geriatrician and stroke physician, one medical social worker, three occupational therapists, six physiotherapists and four speech and language therapists. It was anticipated that a therapy assistant would be interviewed however we did not successfully recruit one. Thirteen healthcare professionals were working directly in Early Supported Discharge, one healthcare professional had worked in Early Supported Discharge until recently but was now working in a different post within stroke and two healthcare professionals were indirectly involved in Early Supported Discharge for example from a governance or secondary prevention perspective. The interviews ranged from 30 to 70 min in length. A description of the participating healthcare professionals is presented in Table 1. A description of Early Supported Discharge team composition by participating hospital is presented in Table 2.

**Table 1.** Description of participants.

No.	Participant ID	Years qualified	Time involved in Early Supported Discharge
1	Occupational Therapist 1	Not recorded	1–5 years
2	Physiotherapist 1	11–15 years	1–5 years
3	Speech and Language Therapist 1	1–5 years	<1 year
4	Speech and Language Therapist 2	6–10 years	1–5 years
5	Physiotherapist 2	1–5 Years	1–5 years
6	Physiotherapist 3	16–20 years	1–5 years
7	Clinical Nurse Specialist	16–20 years	Not recorded
8	Consultant Geriatrician and Stroke Physician	21–25 years	Not recorded
9	Occupational Therapist 2	11–15 years	1–5 years
10	Physiotherapist 4	16–20 years	1–5 years
11	Speech and Language Therapist 3	6–10 years	1–5 years
12	Physiotherapist 5	21–25 years	1–5 years
13	Occupational Therapist 3	16–20 years	6–10 years
14	Speech and Language Therapist 4	6–10 years	1–5 years
15	Physiotherapist 6	16–20 years	6–10 years
16	Medical Social Worker	6–10 years	1–5 years

**Table 2.** Early Supported Discharge team composition by hospital.^7^.

Team	Occupational therapist	Physiotherapist	Speech and language therapist	Therapy assistant	Medical social work	Clinical Nurse Specialist
Recommended WTE	1.0	1.0	1.0	1.0	0.5	0.5
Beaumont Hospital	1.0	1.0	1.0	0.0	1.0	1.0
Cork University Hospital/Mercy University Hospital	1.0	1.0	0.5	0.0	0.0	0.0
University Hospital Limerick	1.0	1.0	0.5	0.0	0.0	0.5
Tallaght University Hospital	1.0	1.0	0.5	0.0	0.5	0.0
University Hospital Galway	1.0	1.0	1.0	1.0	0.2	0.0
Mater Misericordiae University Hospital	1.0	1.0	0.5	1.0	0.5	0.0
Sligo University Hospital	1.0	1.0	0.5	0.0	0.5	0.0
St. Vincent's University Hospital	1.0	1.0	1.0	0.0	0.5	0.0
St. James's Hospital	1.0	1.0	1.0	1.0	0.5	0.0

Five themes were identified:

Un-coordinated development of services, Staff shortages limit the potential of Early Supported Discharge, Limited utilisation of telerehabilitation post COVID-19 pandemic, Families need information and support, and Early Supported Discharge involves collaboration with people after stroke and their families.

### Un-coordinated development of services

Across the interviews, the lack of a national strategy or nationally co-ordinated approach to Early Supported Discharge or the wider stroke pathway was clear. Healthcare professionals described varied funding models for Early Supported Discharge teams, un-coordinated approaches to securing posts/recruitment and retention of staff, disjointed stroke pathways after Early Supported Discharge and inconsistency in core components of Early Supported Discharge across teams.

#### Varied funding models

Across the various sites, staff reported varied funding sources for Early Supported Discharge, with no single dedicated funding stream from national public health service funding. Some healthcare professionals expressed frustration at not being appointed in a full time or permanent capacity despite gathering data while others noted the need to continuously prove within their own hospital the effectiveness of Early Supported Discharge by gathering data for service funders.

*“We have to give a presentation to, like hospital management, to secure funding, you see … even though this has been proven nationally, we still have to keep proving locally that it's, you know, making a difference” (Physiotherapist 4).*

#### Un-coordinated approaches to securing posts/recruitment and retention of staff

Un-coordinated approaches to securing posts and recruitment and retention of staff were frequently reported creating organisational and service delivery challenges. Examples included no cover being provided for staff on annual leave or maternity leave, reductions in staffing or non-replacement of staff. One team was described as understaffed from the outset and in another site a post was vacant for 16 months after a team member resigned. Some participants described making active efforts to manage staffing issues including acute hospital staff in stroke shadowing them in order to cover staff leave and attempts to reach agreements at a local level with hospital Health and Social Care Professionals departments to support unexpected leave. Numerous healthcare professionals were appointed to Early Supported Discharge on a part time basis with the remainder of their post in the acute service. This led to challenges balancing time in both services as demands in the acute service were prioritised over Early Supported Discharge services. “*One of the biggest challenges is all of the others have an inpatient element to their jobs and.…they are pulled very frequently when staffing is a problem which it has been, you know, so the other half of the physiotherapy input is often pulled to cover inpatients” (Physiotherapist 5).*

#### Disjointed stroke pathway after Early Supported Discharge

Healthcare professionals described variable community services to refer people to following the cessation of Early Supported Discharge. Referrals were made to national services such as Acquired Brain Injury Ireland, Headway and the Irish Heart Foundation however the availability of local services varied widely. There was a lengthy waiting list to access a community neurorehabilitation team for one Early Supported Discharge site and an absence of these teams for other healthcare professionals. Primary Community and Continuing Care services typically had long waiting lists and in some cases no availability of certain disciplines. Lack of specialist services was also highlighted specifically related to meeting the needs of a growing cohort, those <65 years of age after stroke. For this group, participants struggled to access specialist services to support return to work, driving and support the person in their role as a parent.

#### Lack of consistency across Early Supported Discharge sites

While Early Supported Discharge criteria were similar across sites, there was some variation in how admission criteria were applied. Some healthcare professionals identified a cohort of people after stroke with impairments that only became apparent in the home or in more challenging environments than the hospital. Therapists described this group as performing well in terms of physical function but having less immediately obvious cognitive, social, emotional or vocational issues. Some participants described their eligibility criteria as broad enough to address these needs while others did not. It was suggested by some that the needs of this cohort may be more suitable for community neurorehabilitation team input. Others considered Early Supported Discharge to be a “*safety net”* for this group. “*It's those walking, you know, the walking wounded that are…..it's, it's just even just makes it more important that someone gets a very comprehensive, detailed, thorough post stroke, work up from a, from a Health and Social Care Professional point of view as much as from a medical point of view” (Occupational Therapist 3).* Most Early Supported Discharge sites offered a “block” of therapy ranging from 6 to 8 weeks; however, several sites were flexible in delaying discharge for some people for various reasons. “*There have been people who've had a recurrent stroke….people who were unwell, you know, say someone got a chest infection and was, like, knocked out for a week or ten days we would maybe give them another little bit. And if someone was waiting the National Rehabilitation Hospital* [specialist rehabilitation hospital in Ireland] *and they really had no date and there is no community services coming any time, we might bridge a little bit, give them a little bit more there as well” (Physiotherapist 1).*

### Staff shortages limit the potential of Early Supported Discharge

Collaborative team working was described as integral to healthcare professional experiences of successful Early Supported Discharge service provision. Notably participants emphasised the important contribution of therapy assistants to the team despite limited assistant posts across teams. Although the importance and benefits of teamwork were reported, a clear pattern of need in terms of team composition, access to therapy assistants, psychologists, medical social workers and clinical nurse specialists was identified across interviews.

#### Collaborative team working is integral to successful Early Supported Discharge

Many positive descriptions of collaboration and teamwork within the Early Supported Discharge teams were reported. Healthcare professionals discussed the benefits of regular meetings to support team communication and discuss goals of people after stroke, their progress and timetables. Collaboration and communication with hospital colleagues was also valued. “*So having that presence* [at hospital multidisciplinary meetings]*, so we're kind of embedded within the acute team to be honest with you, so we are, we have people after stroke flagged to us quite early” (Physiotherapist 3).* Successful collaboration with colleagues in other Early Supported Discharge teams nationally was also described for example sharing learning and experiences when establishing a new service or when teams were adapting practices and procedures during the COVID-19 pandemic. “*So in starting up the team, I suppose we linked in quite closely with local hospitals with established Early Supported Discharge services” (Speech and Language Therapist 3).*

#### Staff shortages

Across all interviews gaps in terms of staffing were described. Access to a psychologist or neuropsychologist to address the cognitive and emotional well-being of people after stroke and their families was commonly identified as a gap. None of the teams in Ireland have a psychologist post as part of the team. Participants described supporting people emotionally and helping them to adjust and cope with losses and changes following a stroke. “*Some of the older cohort as well really struggled with the loss, the loss of independence and loss of role and just the processing experience in general” (Medical Social Worker).* In some cases, the support needed by the person after stroke in adjusting and coping was beyond the scope of practice for the team and access to a psychologist, counsellor or general practitioner was needed. Healthcare professionals identified people after stroke who were in denial about having a stroke, those with fear of recurrent stroke, those struggling with limitations post stroke, those with anxiety and depression, and fearful caregivers as particularly in need of psychological support. However, only one site had access to a psychologist. At one site there was a one year waiting list to access a neuropsychologist while at another site people after stroke could only access a neuropsychologist through a community organisation. Access to medical social workers was also very limited. Several healthcare professionals reported taking on and providing medical social worker input which was described as not ideal. Medical social work intervention ranged from counselling, providing emotional support, engaging with family regarding home care packages, supporting people after stroke regarding welfare issues or decision support mechanisms and assistance in the discharge process. The majority of teams were able to access a nurse to support education of people after stroke and medication management. Two teams had an appointed clinical nurse specialist. Gaps in staffing had various consequences including reconfiguration of services on account of sick leave “*We did actually call the speech and language therapist. She said it was ok while she was off sick. And we were like, is there anything we can do with this patient*” (*Physiotherapist 2*), staff not being backfilled “*Our social worker is actually on maternity leave and she wasn’t backfilled”* (Speech and Language Therapist 1), limits on accepting new referrals or slower movement of service users through the service.

#### Limited access to therapy assistants

Three sites had a therapy assistant as part of the Early Supported Discharge team; however, only two were in post with one post vacant. Healthcare professionals considered them to be an integral part of the team, a valuable addition, and a “*central figure for the person after stroke”* (Speech and Language Therapist 4). “*Every team should have one…our therapy assistant is incredible with patients, in teaching me but just really delivering that intensive therapy”* (Speech and Language Therapist 2). Therapy assistants increased team capacity, enabled the intensity of rehabilitation to be increased, facilitated healthcare professionals to concentrate on people after stroke with complex needs and provided an opportunity for those living outside of the locality to access the service. Therapy assistants were responsible for implementing elements of rehabilitation programmes with guidance and supervision from therapists along with providing feedback on participation and progression in rehabilitation of the person after stroke. Despite the identified benefits only three of the nine sites had a therapy assistant post. Participants working in teams without an assistant post identified a need to have one and some sites had submitted business cases to secure this role.

### Limited utilisation of telerehabilitation post COVID-19 pandemic

Healthcare professionals described the impact of the COVID-19 pandemic on the delivery of Early Supported Discharge, the adaptations made to ensure service provision continued as well as the impact of the COVID-19 pandemic on them. Three teams were established during the COVID-19 pandemic. During the various COVID-19 waves, all bar one team implemented telerehabilitation exclusively or alongside face-to-face service provision. However by the time of interview almost all had abandoned telerehabilitation and most participants emphasised the advantages of face-to-face therapy in the home environment over telerehabilitation.

#### Rapid adoption of telerehabilitation at the outset of the COVID-19 pandemic

The introduction of telerehabilitation was a major change to the delivery of Early Supported Discharge services during the COVID-19 pandemic. Initial challenges included finding computers that were able to support the technology needed to delivery telerehabilitation, accessing assistance from information technology colleagues and navigating the many platforms that people after stroke could access. Healthcare professionals had mixed opinions about telerehabilitation. Reported benefits included increased capacity due to less travel time and being able to successfully carry out interventions that they initially didn’t think would be feasible. Challenges in using telerehabilitation for Early Supported Discharge included people after stroke having connectivity or digital access limitations, digital poverty, lack of digital skills, the need for family to support technology use, and privacy limited by the presence of family.

#### Limited use of telerehabilitation post COVID-19 pandemic

Only one healthcare professional reported that they had continued to regularly incorporate telerehabilitation into interventions with people after stroke at the time of interview when almost all COVID-19 restrictions were lifted. Some teams continued to use telerehabilitation to enable access to people after stroke that lived outside of the locality to access the service. “*I’m still taking people that are just purely telehealth if they fall outside of my catchment to drive to them and in the absence of nothing else” (Occupational Therapist 2).*

Some participants questioned the effectiveness of telerehabilitation. “*I feel like we need to remind ourselves that all the evidence based and everything we're going on is based on face-to-face therapy, and until that evidence comes out that that* [telerehabilitation] *is equal, then I think where possible we should still be delivering an evidence based service which is face-to-face” (Occupational Therapist 3)*. Furthermore, healthcare professionals expressed a preference to develop a therapeutic relationship within the home in a face-to-face capacity and to be “*hands on”* (*Physiotherapist 6*) with people after stroke. “*It's all about building relationships and trust and you can't do that through being online you know” (Medical Social Worker).* Certain interventions were difficult to complete through an online medium for example interventions for shoulder pain, visual perceptual deficits and cognitive rehabilitation. Other benefits of working directly with people after stroke face-to-face in their own homes were people after stroke were more empowered at home, and therapists could observe real life interactions and situations unfolding in the home allowing difficulties to be revealed. The home had everyday tools and objects that could be integrated into functional rehabilitation and healthcare professionals viewed the home as an optimal site for rehabilitation.

### Families need information and support

Healthcare professionals highlighted the important contribution of family members to the success of Early Supported Discharge and the need for healthcare professionals to support people after stroke and family members and provide them with sufficient information.

#### Important contribution of families to rehabilitation

All healthcare professionals discussed the role of the family during Early Supported Discharge. Family involvement in therapy sessions was described as a motivator for people after stroke and examples of spouses, adult children and children under 18 years being involved in therapy were provided. Healthcare professionals described family members supporting continued rehabilitation programmes in the absence of the healthcare professional, providing collateral information and supporting goal setting particularly if the person after stroke had cognitive issues. Speech and language therapists described educating and supporting family members, managing their expectations, providing strategies and addressing unhelpful communication styles.

#### Families have information and support needs

Healthcare professionals reported people after stroke and family members’ understanding of Early Supported Discharge varied at the point of hospital discharge and for some families there was concern about early discharge from acute services. “*There's been an odd occasion with the family member when they hear the word early, they get a bit anxious that they're going home early and then it's just stressed that they're medically fit for discharge. You know, they're not needing any more medical treatment, so it's more education around the service” (Physiotherapist 4).* One team reported they were considering changing their team name to remove reference to “Early” as it was perceived to be the only word heard by family members.

Information was central to the acceptance of Early Supported Discharge. Some people after stroke and families agreed to the service as a means of leaving the hospital but did not understand it, perceiving it as a support service. There was an element of distrust that such a service could exist within the Irish health service. Some people after stroke and family members preferred inpatient rehabilitation however the presence of the COVID-19 pandemic increased openness to Early Supported Discharge due to inpatient visitor restrictions. “*It's kind of been the opposite where people are mad to get home, especially with COVID-19 and visiting restrictions and that they'd be like, “No, I'm not going to rehabilitation, but I want to go home” and then they hear about our team that can come in and see them in their home and they're absolutely delighted then” (Physiotherapist 4).*

Educating families about stroke was part of the healthcare professional role however visiting restrictions in the hospital due to COVID-19 meant families had a lot of questions and felt ill-informed. Restrictions on visiting in the hospital due to COVID-19 meant families were unable to see the person after stroke during or prior to their discharge and may not have been as sufficiently prepared as in pre-COVID-19 times. “*And then they didn't see them* [while in hospital] *so they* [the family] *didn't know maybe how the stroke had affected them” (Physiotherapist 5).*

### Early Supported Discharge involves collaboration with people after stroke and their families

Healthcare professionals described working in collaboration with people after stroke and their families or carers. Goal setting was central to collaboration and participants considered how best to support the person after stroke to engage in goal setting. Collaboration during rehabilitation was described in terms of the use of meaningful activities during rehabilitation.

#### Goal setting is central to collaboration

Most participants described goal setting with the person after stroke to guide the focus of rehabilitation. Some participants commented that Early Supported Discharge worked so well because it focused on goals that were pertinent to the person's real life. “*The person can say to you, “I want to walk up to the local shopping centre and I want to go to SuperValu* [Irish supermarket]*”* *like it's so real life that, you know, they can have very real goals and we can actually work towards them obviously, depending on what the person wants” (Occupational Therapist 1).* Several participants described thoughtful consideration of how to enable the person after stroke participate in collaborative goal setting. It was acknowledged that goal setting could be hard for some people after stroke as they struggled to contextualise where they were in their recovery journey. Several participants described goal setting as a process best engaged in after the first visit as people after stroke need time to adjust to being at home and process what had happened to them. “*So that they have a bit of time to adjust to home and to see what are the immediate things that you have to get them to be able to do” (Physiotherapist 1).* The first meeting was seen as an opportunity to meet the person after stroke, see how they were managing, explain the service and prompt the person after stroke to start thinking about goals.

#### Collaboration during delivery of therapy

The content of rehabilitation or interventions employed by therapists frequently was delivered via activities that were meaningful to the person after stroke and identified during goal setting. These activities were described by one healthcare professional as “*wild and wonderful” (Physiotherapist 6)* and ranged from clay pigeon shooting, returning to golf, going to a coffee shop, farming activities and facilitating conversational opportunities to have that social connection with friends in the pub. When delivering rehabilitation, several healthcare professionals described being aware of the service users’ comfort level with taking risk. People after stroke were seen by some participants to range from being overly risk averse to taking undue risks. Positive and successful risk taking during rehabilitation aimed for by therapists in their collaborative practice with the person after stroke was reported as empowering for the service user. “*“*C*an I walk outside on my own?” I was like, well, have you already done that? And they're like, yes, I have.“I* just want to know if really I'm allowed?*”* I suppose, I believe it from a therapy perspective there there's a balance. Like you can't restrict people too much. There is always a balance in terms of allowing them to take a risk” (*Physiotherapist 5*).

## Discussion

This study explored healthcare professionals’ experiences of the development and delivery of an Early Supported Discharge service in Ireland. We identified five key themes; Un-coordinated development of services (theme 1), Staff shortages limit the potential of Early Supported Discharge (theme 2), Limited utilisation of telerehabilitation post COVID-19 pandemic (theme 3), Families need information and support (theme 4), and Early Supported Discharge involves collaboration with people after stroke and their families (theme 5).

Healthcare professionals described gaps in staffing resulted in the curtailment of Early Supported Discharge services. Healthcare staffing shortages not only limits the capacity to deliver quality care but can also harm staff well-being.^20^ Given the planned expansion of Early Supported Discharge services in Ireland,^6^ retention and recruitment of healthcare professionals needs to be prioritised. Access to psychology services was described as particularly limited. Internationally, challenges also exist in accessing psychology services for people after stroke,^8,21^ for example 66.9% of community-based multidisciplinary stroke teams (including Early Supported Discharge) in England, Wales and Northern Ireland have access to psychology.^8^ However, the situation in Ireland is markedly worse as only 5% of people after stroke in Ireland had access to psychological services in 2020.^6^

Participants valued therapy assistants as they increased therapy intensity and service capacity. Therapy assistants as members of an Early Supported Discharge team aligns with international best practice is endorsed by the Irish National Stroke Strategy^6^ and recommended as a core team member by the National Clinical Guideline for Stroke for the United Kingdom and Ireland.^5^ Our findings reflect recent qualitative research with healthcare professionals in the United Kingdom (n = 117) where therapy assistants were described as having a key role to play in the team and development of an interdisciplinary skillset among therapy assistants helped manage capacity issues.^9^ Importantly, this study highlighted the need to empower and promote the autonomy of assistant staff through appropriate supervision and upskilling.^9^ Other research has confirmed that therapy assistants can improve capacity and efficiency^22^ however to date they may be underutilised within community settings.^23^ Further research is needed to quantify the benefits and cost implications of therapy assistants as part of Early Supported Discharge teams.

Integration of existing services, including Early Supported Discharge and community stroke rehabilitation, forms the basis of the National Health Service England Integrated Community Stroke Service model.^24^ Similarly, in Ireland, Sláintecare emphasises the delivery of person-centred care and integrated services.^6^ However, we found that access to local and national services following discharge from Early Supported Discharge is variable.

The development and implementation of telehealth was expedited by the COVID-19 pandemic.^25^ People after stroke who received telerehabilitation have similar outcomes compared to those that received face-to-face therapy.^26^ Our study highlighted benefits and challenges to delivering services through telerehabilitation including reduced travel and increased capacity while a digital divide amongst people after stroke, connectivity issues and privacy issues were some of the challenges reported. Telerehabilitation is assumed to be less expensive however information on its cost-effectiveness is limited.^26^ Notably despite the reported benefits only one participant reported regularly using telerehabilitation at the time of interview. Previous research has shown that people after stroke and healthcare professionals value the home environment as an optimal setting for rehabilitation.^4^ In this study, healthcare professionals felt that telerehabilitation limited therapeutic alliance and rehabilitation was limited by the lack of access to the home environment. Future research should focus on developing and evaluating models of technology use in Early Supported Discharge in terms of equity of access, acceptability, effectiveness and cost-effectiveness.

Healthcare professionals in our study felt that inclusion and support of family members was important. Other studies have also endorsed the need for caregiver involvement and preparedness.^27,28^ Caregiver burden after stroke is well documented^29^ and the transition from hospital to home presents specific challenges for caregivers.^30^ Additionally, participants felt caregiver challenges were exacerbated by visiting restrictions during the COVID-19 pandemic. Research on hospital visiting restrictions, not specific to stroke, found restrictions were an additional stressor for both the patient and relatives but affected relatives more.^31^

Healthcare professionals in this study identified the need to address challenges affecting younger people after stroke. There is an increase in the proportion of strokes occurring among those younger than 70 years of age^1^ and there is an economic burden associated with stroke among those of working age.^32,33^ Unmet needs previously identified amongst this population included social participation,^34^ financial,^34^ returning to work^35^ and using contraception after stroke.^35^ Greater research focus on whether Early Supported Discharge is adequately meeting the needs of younger people after stroke and caregivers, partners or family members to ensure that services meet their needs in a timely manner is warranted.

This study has a number of strengths, and we recruited participants from each of the Early Supported Discharge teams thus providing an overview of service delivery across Ireland. The conduct and reporting of this study adhered to the Consolidated Criteria for Reporting Qualitative Research (COREQ) guidelines.^13^ However, all healthcare professionals interviewed were based in Early Supported Discharge teams in Ireland therefore findings may not reflect context specific factors in other countries. As only one medical social worker, consultant geriatrician and stroke physician and clinical nurse specialist, respectively, were interviewed; additional participants from these disciplines may have yielded further insights. Furthermore, while participants endorsed therapy assistants as members of the team we were unsuccessful in recruiting a therapy assistant to the study.

Health professionals’ experiences of Early Supported Discharge after stroke in Ireland have been explored in this study. Managers and funders of Early Supported Discharge teams should address staff recruitment and retention to prevent service shortages and should ensure consistent access to psychology services. Services should continue to work closely with families and address their information and support needs during Early Supported Discharge. Future research on how telerehabilitation can optimally be deployed and the effectiveness of input and involvement of therapy assistants in Early Supported Discharge is needed.

**Clinical messages**

1. Access to psychological services for people after stroke is an enduring problem
2. Healthcare providers believe therapy assistants can support Early Supported Discharge team capacity
3. Family members should be included and supported during Early Supported Discharge
